# 4F Decreases IRF5 Expression and Activation in Hearts of Tight Skin Mice

**DOI:** 10.1371/journal.pone.0052046

**Published:** 2012-12-14

**Authors:** Hao Xu, John G. Krolikowski, Deron W. Jones, Zhi-Dong Ge, Paul S. Pagel, Kirkwood A. Pritchard, Dorothée Weihrauch

**Affiliations:** 1 Division of Pediatric Surgery, Department of Surgery, Children’s Research Institute, Milwaukee, Wisconsin, United States of America; 2 Division of Anesthesiology, Department of Surgery, Children’s Research Institute, Milwaukee, Wisconsin, United States of America; 3 The Clement J. Zablocki Veterans Affairs Medical Center, Milwaukee, Wisconsin, United States of America; University of Otago, New Zealand

## Abstract

The apoAI mimetic 4F was designed to inhibit atherosclerosis by improving HDL. We reported that treating tight skin (Tsk^−/+^) mice, a model of systemic sclerosis (SSc), with 4F decreases inflammation and restores angiogenic potential in Tsk^−/+^ hearts. Interferon regulating factor 5 (IRF5) is important in autoimmunity and apoptosis in immune cells. However, no studies were performed investigating IRF5 in myocardium. We hypothesize that 4F differentially modulates IRF5 expression and activation in Tsk^−/+^ hearts. Posterior wall thickness was significantly increased in Tsk^−/+^ compared to C57Bl/6J (control) and Tsk^−/+^ mice with 4F treatment assessed by echoradiography highlighting reduction of fibrosis in 4F treated Tsk^−/+^ mice. IRF5 in heart lysates from control and Tsk/+ with and without 4F treatment (sc, 1 mg/kg/d, 6–8 weeks) was determined. Phosphoserine, ubiquitin, ubiquitin K^63^ on IRF5 were determined on immunoprecipitates of IRF5. Immunofluorescence and TUNEL assays in heart sections were used to determine positive nuclei for IRF5 and apoptosis, respectively. Fluorescence-labeled streptavidin (SA) was used to determine endothelial cell uptake of biotinylated 4F. SA-agarose pulldown and immunoblotting for IRF5 were used to determine 4F binding IRF5 in endothelial cell cytosolic fractions and to confirm biolayer interferometry studies. IRF5 levels in Tsk^−/+^ hearts were similar to control. 4F treatments decrease IRF5 in Tsk^−/+^ hearts and decrease phosphoserine and ubiquitin K^63^ but increase total ubiquitin on IRF5 in Tsk^−/+^ compared with levels on IRF5 in control hearts. 4F binds IRF5 by mechanisms favoring association over dissociation strong enough to pull down IRF5 from a mixture of endothelial cell cytosolic proteins. IRF5 positive nuclei and apoptotic cells in Tsk^−/+^ hearts were increased compared with controls. 4F treatments decreased both measurements in Tsk^−/+^ hearts. IRF5 activation in Tsk^−/+^ hearts is increased. 4F treatments decrease IRF5 expression and activation in Tsk^−/+^ hearts by a mechanism related to 4F’s ability to bind IRF5.

## Introduction

**Figure 1:**
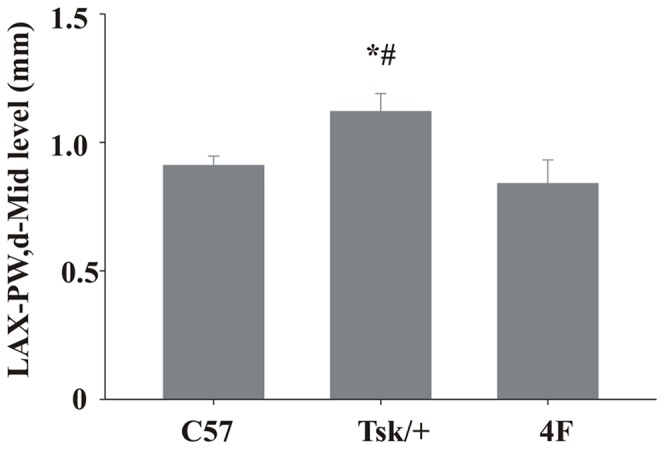
Echocardiography of hearts from Tsk/+ mice with and without 4F treatment and C57Bl/6J mice as control mouse. Posterior wall thickness was significantly improved by 4F treatment in the Tsk/+ mice (p<0.05).

Scleroderma is an autoimmune disease that is characterized by marked increases in fibrosis of the skin and internal organs in conjunction with enhanced apoptosis [1]. The tight skin (Tsk^−/+^) mouse is a murine model of systemic scleroderma that replicates many of the features observed in patients suffering from this disease [2]. Previously, we showed that Tsk^−/+^ mice have impaired endothelial vasodilatation and angiogenic responses to VEGF that coincided with marked increases in angiostatin [3]. We also showed that the matrix isolated from the hearts of Tsk^−/+^ mice stimulated endothelial cells (EC) to transition into fibroblasts and assume a more fibrotic phenotype [1]. Thus the extracellular matrix appears to play an important role in the mechanisms responsible for the vascular abnormalities in these mice. 4F is an apoA-I mimetic that was designed to improve high-density lipoprotein (HDL) function and has been shown to decrease proinflammatory HDL levels [4], [5]. Our laboratory has observed that not only do 4F treatments inhibit vascular disease in Tsk^−/+^ mice but even appear to delay the onset of heart failure in Tsk^−/+^ mice (Figure 1).

Although there is much support for the idea that 4F inhibits atherosclerosis by improving HDL function [6], the mechanism by which 4F decreases inflammation has not been identified completely. Several mechanisms have been proposed including improvements in reverse cholesterol transport [7], formation of preβ HDL with improved HDL-dependent cholesterol efflux [4] and even greater affinity for oxidized rather than native lipids [8]. During the course of our studies for our report [1], [3], we found the anti-inflammatory effects of 4F on the Tsk^−/+^ mice to be so profound that it was difficult to attribute all of the effects solely to improvements in HDL function. While the published mechanisms may play important roles in the prevention of atherosclerosis none seemed sufficient for explaining why 4F protects vascular function and decreases inflammation in Tsk^−/+^ mice, an extreme murine model of autoimmune disease. To better understand this paradox, we began to investigate alternative mechanisms for 4F protecting the myocardium in Tsk^−/+^ mice.

IRF5 is a transcription factor that has been called a “master switch” in immunity because it directs responses in macrophages and other inflammatory cells [9], [10] and has been implicated as a key mediator of apoptosis [11], [12]. Although much research has defined IRF5 cell biology in immune cells, little if any research is available concerning the role of IRF5 in the myocardium. In the current study we examine the effects of 4F on IRF5 in the hearts of Tsk^−/+^ mice and test the hypothesis that 4F decreases myocardial inflammation in the Tsk ^−/+^ mice by inhibiting IRF5.

## Materials and Methods

### Peptide Synthesis

4F (Ac-DWFKAFYDKVAEKFKEAF-NH2) and biotin-labeled 4F (B-4F, biotin-labeled DWFKAFYDKVAEKFKEAF-NH2) were synthesized by FMOC techniques using all D-amino acids (GenScript USA Inc, Piscataway, NJ). B-4F was synthesized in the same way but with one more cycle of synthesis with FMOC-Lys(biotinyl-ε-aminocaproyl)-OH. Peptides were synthesized, purified by HPLC (>95%), and the purity confirmed by mass spectrometry by GenScript USA Inc [3].

### Mice and Experimental Treatments

All experimental procedures and protocols involving mice were reviewed and approved by the Institutional Animal Care and Use Committee (IACUC) of the Medical College of Wisconsin (AUA#512). They also conformed to the rules in the *Guiding Principles in the Care and Use of Animals* published by the American Physiologic Society and the *Guide for the Care and Use of Laboratory Animals* published by OLAW. Male Tsk^−/+^ and C57Bl/6J mice (6–8 weeks of age) were purchased from Jackson Laboratories (Bar Harbor, ME). Three groups of mice were studied; C57Bl/6J, Tsk^−/+^, and Tsk^−/+^ mice treated with 4F (1 mg/kg/d, sc) as published previously [3].

### Transthoracic Echocardiography

Tsk^−/+^ and C57BL/6J mice (n = 6) were anesthetized by isoflurane inhalation (1.50%) and oxygen. They were place on a warming platform supine position. A VisualSonics Vevo 770 high-resolution imaging system (Toronto, Canada) equipped with a 30-MHz transducer (Scanhead RMV 707) was used to perform the transthoracic echocardiography. Two-dimensional guided M-mode LV dimensions were measured [13] and posterior wall thickness was analyzed.

### IRF5 Expression

Hearts harvested from C57Bl/6J, Tsk^−/+^ and 4F-treated Tsk ^−/+^ mice were snap-frozen and stored at −80°C for subsequent analysis. Frozen hearts were pulverized in a stainless steel mortar and pestle bathed in liquid nitrogen. The frozen powder was dissolved and homogenized in lysis buffer [MOPS pH 7.0, 20 mM; EGTA 2 mM; EDTA 5mM; NaF 30 mM; Protease Inhibitor Cocktail (Complete Mini Roche, Roche, Madison WI); Phosphatase Inhibitor Cocktail (Sigma P5726)]. Lysate proteins were quantified using BCA (bicinchoninic acid) reagent (Pierce, Rockford, IL). An aliquot of 40 µg lysate protein was diluted in Laemmli sample buffer [3], [14] containing mercaptoethanol and heated to 95°C for 5 minutes, loaded onto SDS page gels (4–15%) and proteins separated by electrophoresis as described [1]. Proteins were transferred onto nitrocellulose membranes and blocked in Tris buffered saline (TBS) containing Tween (0.05%, TBS-T) and 10% non-fat dry milk for one hour and incubated with rabbit anti-IRF5 antibody (Protein-Tech, Chicago, IL; 1∶800 dilution in TBS). The next day blots were washed two times, 5 minutes each time, in TBS-T and incubated with a HRP-conjugated anti-rabbit antibody (Santa Cruz Biotechnologies, Santa Cruz, CA, 1∶5000 dilution) for one hour. Levels of IRF5 were obtained by scanning autoradiograms that were generated on Gene Mate film (BioExpress, Kayville, UT) with ECL plus Western Blotting Reagents (GE Healthcare, Piscataway, NJ) and developed with Kodak reagents (Rochester, NY). Images of band densities in the autoradiograms were captured using Photostudio software (ArcSoft, Fremont, CA) and a flatbed scanner (Canon, Model N12204 Lake Success, NY). Band densities were analyzed using Image J software (NIH, Bethesda, MD) [3].

### IRF5 Post Translational Modifications

To determine the effects of strain and 4F treatments on the activation state of IRF5 we immunoprecipitated IRF5 from heart homogenates and examined changes in phosphoserine and ubiquitination on IRF5 using previously published protocols [14], [15]. Briefly, heart homogenates were precleared with agarose beads for 1 hour followed by addition of anti-IRF5 antibody (Santa Cruz Biotechnologies) to the homogenates (500 µg in 0.5 ml) at 5 µg/100 µl (final concentration). Samples were incubated for 16 h at 4°C with end-over-end mixing. A 50 µl aliquot of a 50% slurry of Protein A agarose beads (Santa Cruz Biotechnologies) was added and incubated for 2 h with end-over-end mixing to pull-down immunocaptured IRF5. The beads were isolated by centrifugation and washed 3 times (3 minutes each time) in PBS. IRF5 was released from the beads with Laemmli sample buffer (Bio-Rad, Hercules, CA) containing mercaptoethanol and heating to 95°C for 5 min. IRF5 was separated from co-precipitated proteins by SDS-PAGE (4–15% Tris·HCl Criterion gels, Bio-Rad) and the proteins transferred onto nitrocellulose membranes. Membranes were blocked in 10% non-fat dry milk and then incubated in 2% non-fat dry milk containing polyclonal rabbit anti-phosphoserine (Antibodies-Online, Atlanta, GA, 1∶200 dilution), monoclonal mouse anti-ubiquitin (Santa Cruz Biotechnologies, 1∶200 dilution) or monoclonal mouse anti-K^63^ ubiquitin (Enzo, Farmingdale, NY, 1∶500 dilution) overnight at 4°C. The next day, the membranes were gently washed and incubated with the HRP-conjugated donkey anti-rabbit antibody (1∶5,000, Santa Cruz Biotechnologies). Autoradiograms were developed; images of bands captured and densities analyzed as described above.

### Measurements of IRF5 Positive Nuclei

Hearts were harvested from mice, flash frozen and imbedded in OCT and cut into 10 µm sections. Frozen sections were fixed for 10 minutes in PBS containing 1% paraformaldehyde. Cell membranes were permeabilized by incubation in PBS containing 0.5% Triton-X for 5 min at room temperature. The sections were washed once in PBS (5 min) and incubated in PBS containing mouse monoclonal anti-IRF5 antibody (Santa Cruz, 1∶50 concentration) for 30 min at 37°C. Unbound anti-IRF5 antibody was washed free from the sections with PBS and the slides incubated with the appropriate Alexa 488-conjugated secondary antibody (Invitrogen, Carlsbad, CA) for 30 min at 37°C. The slides were washed two times with PBS and then incubated with TO-PRO 3™ for 3 min at room temperature (1 µg/ml, Invitrogen) to visualize cell nuclei. Sections were mounted in aqueous mounting media and analyzed by confocal microscopy [16], [17].

### Measurements of Apoptotic Cells

Hearts were harvested from mice, flash frozen and imbedded in OCT and cut into 10 µm sections. A TUNEL assay was performed on the sections using an ApopTag Plus Fluorescein labeled kit (Millipore, Billerica, MA, Cat# S7111) as per the manufacturer’s instructions. Briefly, the free 3'OH DNA termini were labeled *in situ* with an anti-digoxigenin antibody conjugated to fluorescein. Nuclei were stained with TO-PRO 3™ for 3 minutes (1 µg/ml, Invitrogen). Slides were mounted and analyzed by confocal microscopy (Nikon Eclipse TE2000-U, EZ-C1-2 10 software, Nikon, Melville, NY). Images were captured using 488 nm excitation for fluorescein and 633 nm for nuclear labeling. Intensity was measured using Nikon Element imaging software (Nikon) [1].

### Cellular Uptake of B-4F

Human umbilical vein endothelial cells (EC; Lonza, Walkersville, MD) were incubated in EGM complete media containing 4F or B-4F (10 and 50 µg/ml) for 4 hours. The EC cultures were washed with PBS, fixed in PBS containing 1% paraformaldehyde, and permeabilized with PBS containing 0.5% Triton-X. The permeabilized sections were incubated with fluorescein-conjugated streptavidin (1 µg/ml) for 30 minutes followed by 3 PBS washes. The fixed and fluorescently labeled slides were mounted in aqueous mounting media and sections inspected and analyzed by confocal microscopy [18].

### IRF5 and 4F Interactions

Biolayer Interferometry (BLI): Protein-protein interactions were determined by BLI using an Octet Red 96™ interferometer (ForteBio, Inc, Menlo Park, CA). IRF5 was purchased (GenScript USA Inc) and labeled with biotin (cat no. 21329, Thermo Fisher Scientific, Rockford, IL) at a ratio of 1∶1, which is optimal for binding and packaging target proteins onto SA biosensors (MR18-0009, ForteBio) for studying protein-protein interactions. Biotin-labeled IRF5 (B-IRF5) was loaded onto SA-biosensors in PBS for 15 minutes to ensure saturation. The B-IRF5-loaded SA biosensors were incubated in PBS containing 4F (20 µg/ml) for 10 minutes and association measured by following increases in wavelength interference. Next the loaded biosensors were incubated in PBS alone for 10 minutes to measure dissociation of 4F from B-IRF5 by following decreases in wavelength interference [19].

B-4F Pulldown Assays: To determine whether 4F interacts with IRF5 in the presence of cystosol proteins, we performed 4F pulldown assays on EC cytosolic fractions spiked with recombinant IRF5. Briefly, cytosolic EC fractions were precleared with SA agarose beads (Cat#15942-050, Invitrogen) for 30 minutes at 4°C. IRF5 interactions were enhanced by adding recombinant IRF5 (2% wt/vol, Genscript USA Inc.) to the endothelial cytosolic fractions. 4F and B-4F were added to the cytosolic fractions and incubated for 3 hours at 4°C. SA agarose beads were added and the samples mixed end-over-end overnight at 4°C. The following day the SA agarose beads were isolated by centrifugation. The proteins associated with B-4F were released from the beads with Laemmli buffer and separated by SDS-PAGE (4–15%) as above. IRF5 levels in the pulldown samples were determined by autoradiography of immunoblots as above [20].

### Statistical Analysis

Statistical analysis of data within and between groups was performed with analysis of variance (ANOVA) for repeated measures followed by Bonferroni’s modification of Student’s t-test. The null hypothesis was rejected when P<0.05. All data are expressed as mean ± standard error of the mean (SEM).

## Results

### Echocardiography

To measure an improvement of heart function we measured posterior wall thickness by echocardiography. Posterior wall thickness was significantly higher in the Tsk^−/+^ mice compared to C57Bl/6J and Tsk^−/+^ mice treated with 4F (p<0.05) (Figure 1).

### Myocardial IRF5 Expression

**Figure 2:**
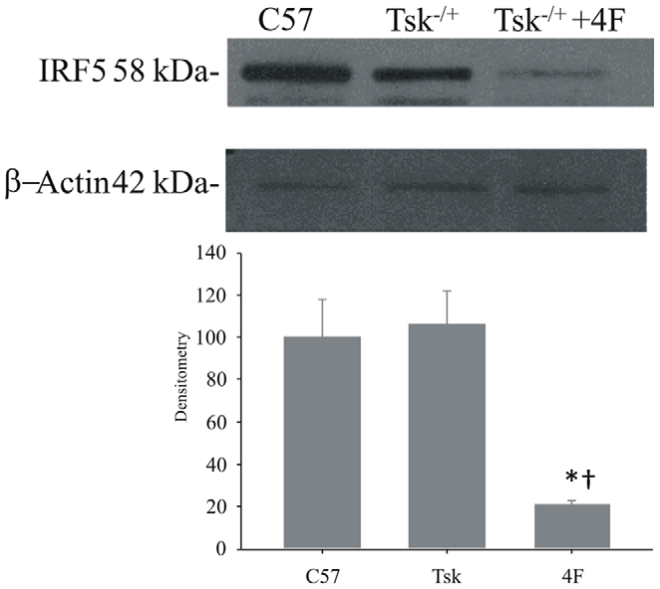
Western blot analysis of myocardial IRF5 protein expression. These blots show that IRF5 expression is unchanged in Tsk^−/+^ myocardium when compared to C57Bl/6J myocardium. 4F treatments (s.c. 1 mg/kg/d for 6weeks) however, markedly decrease IRF5 expression in Tsk^−/+^ myocardium.

IRF5 expression in Tsk^−/+^ hearts was essentially the same as in control hearts (Figure 1, lane 2 vs. lane 1). Interestingly, 4F treatments markedly decreased IRF5 expression in Tsk^−/+^ hearts compared with the levels in hearts isolated from untreated Tsk^−/+^ mice (Figure 2, lane 3 vs. lane 2). Image analysis of band densities indicate that 4F treatments reduced IRF5 in Tsk^−/+^ hearts by 78%.

### Myocardial IRF5 Phosphoserine and Ubiquitin

**Figure 3:**
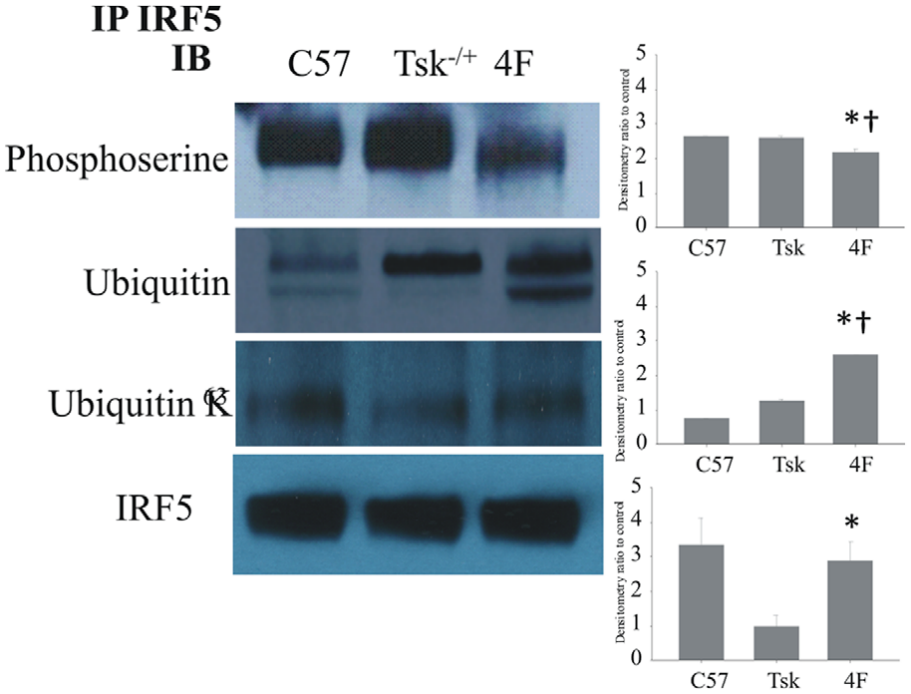
Western blot analysis of myocardial IRF5 immunoprecipitates. Western blot analysis of IRF5 immunoprecipitates from whole heart lysates shows that phosphoserine levels on IRF5 were essentially the same in Tsk^−/+^ hearts as the levels on IRF5 in C57BL/6J hearts. 4F treatments reduced phosphoserine levels on IRF5 in Tsk^−/+^ hearts compared with the levels on IRF5 in hearts from Tsk^−/+^ and C57BL/6J mice. Total ubiquitin on IRF5 was upregulated in Tsk^−/+^ hearts and 4F treatments of Tsk^−/+^ mice, significantly increased total ubiquitin levels on IRF5 in the Tsk^−/+^ hearts compared with the levels on IRF5 in C57BL/6J hearts (* = p<0.05, n = 4). Finally, K^63^ ubiquitin on IRF5 in Tsk^−/+^ hearts is decreased compared to the levels on IRF5 in C57Bl/6J myocardium. 4F treatments of Tsk^−/+^ mice increased K^63^ ubiquitin on IRF5 in Tsk^−/+^ hearts with the levels on IRF5 from treated Tsk^−/+^ mice being the highest when compared with levels in C57BL/6J and Tsk^−/+^ myocardium.

IRF5 activation is regulated by serine phosphorylation and ubiquitination [21], [22]. Phosphoserine levels on IRF5 in Tsk^−/+^ hearts were essentially the same as the levels on IRF5 in C57BL/6J hearts (Figure 3A). In contrast, the phosphoserine levels on IRF5 in 4F-treated Tsk^−/+^ hearts were decreased by about 15% when compared with the levels on IRF5 in C57BL/6J and the untreated Tsk^−/+^ hearts. Interestingly, total ubiquitin on IRF5 was increased in 4F-treated Tsk^−/+^ hearts compared with the levels on IRF5 in C57BL/6J hearts (Figure 3B). The ubiquitinated lower band (Figure 3B, lane 3) may represent an inactive degradation product of IRF5 as the molecular weight of this band is notably less than the molecular weight of IRF5 in untreated Tsk^−/+^ hearts (Figure 3B, lane 2). These post-translational changes in IRF5 coincided with marked decreases in IRF-5 nuclear localization (Figure 3) suggesting that the increase in ubiquitin on IRF5 in 4F-treated Tsk^−/+^ mice may have resulted in IRF5 degradation rather than nuclear translocation. Although ubiquitination is reported to play a role in IRF5 translocation to the nucleus [23], ubiquitin K^63^ levels on IRF5 were actually decreased in hearts of Tsk^−/+^ mice and increased in hearts of 4F-treated Tsk^−/+^ mice (Figure 3C). These data suggest that IRF5 is activated in Tsk^−/+^ hearts and inactivated in 4F-treated Tsk^−/+^ hearts. Our observation that phosphoserine decreased and total ubiquitin increased on IRF5 in 4F-treated Tsk^−/+^ hearts relative to the levels on IRF5 in untreated Tsk^−/+^ hearts is consistent with this possibility. Taken together these changes suggest that 4F treatments inactivate IRF5 and increase its degradation in Tsk^−/+^ hearts.

### Myocardial IRF5 Positive Nuclei and Apoptotic Cells

**Figure 4:**
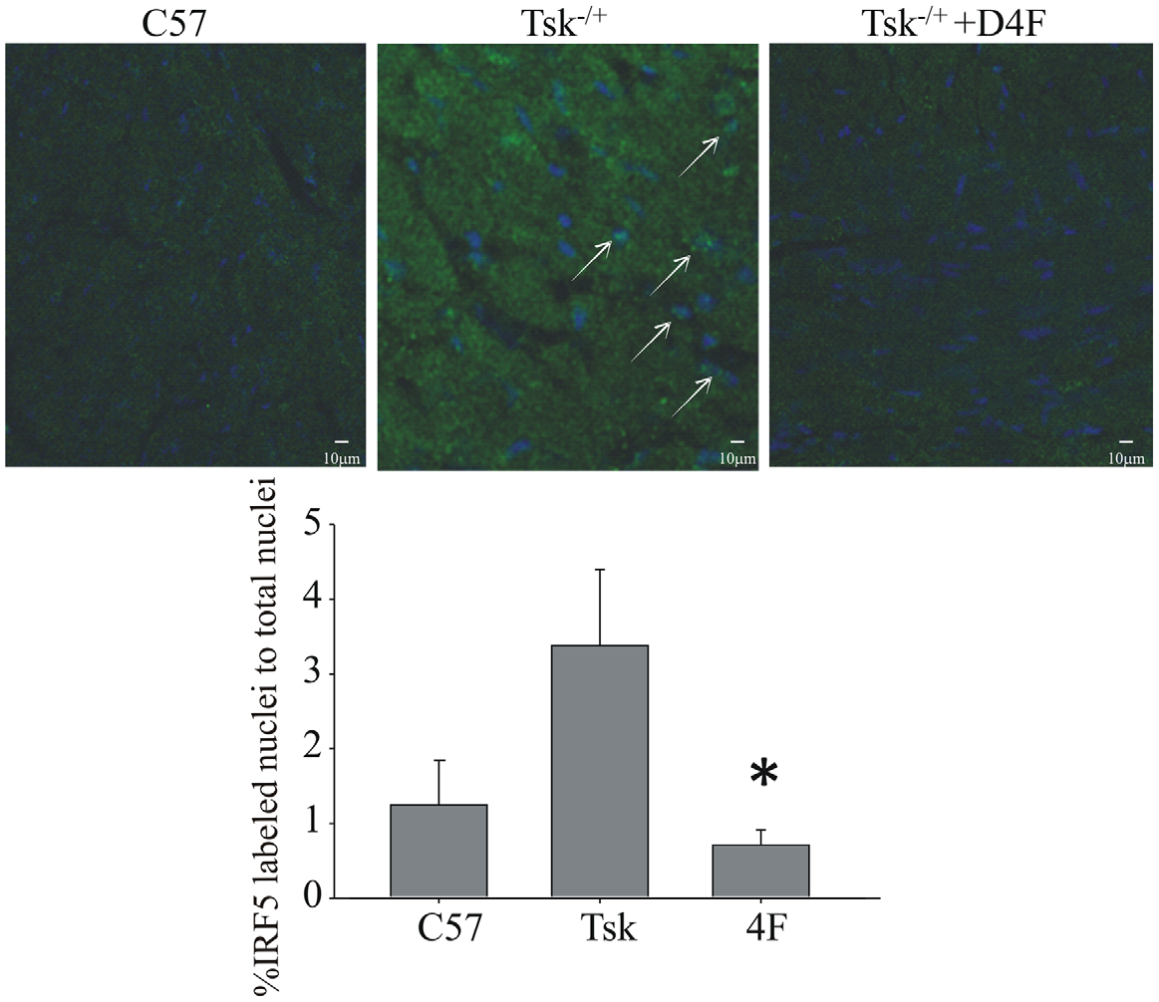
Immunohistochemistry for IRF5 depicted significantly more nuclear labeling in Tsk^−/+^ hearts compared to C57BL/6J hearts and hearts from Tsk^−/+^ mice treated with 4F. The bar graph shows the ratio between green IRF5 labeled nuclei to total nuclei number (p<0.05, n = 3).

**Figure 5:**
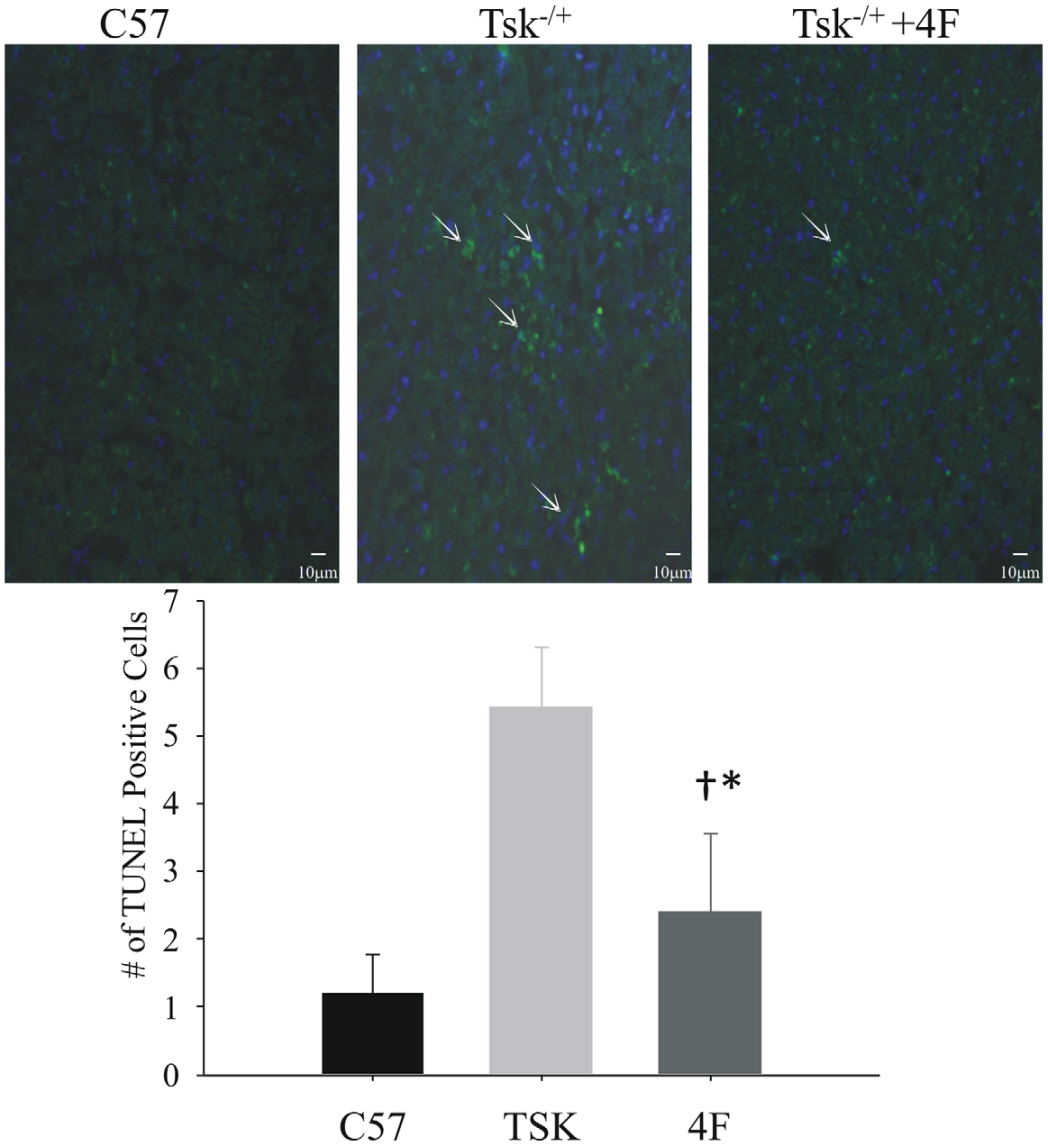
Apoptosis in the myocardium of C57BL/6J, Tsk^−/+^ mice with and without 4F treatments. The number of apoptotic cells in C57BL/6J hearts is low. The number of apoptotic cells in the myocardium of Tsk^−/+^ mice is increased compared with the levels in the hearts of C57BL/6J mice and hearts from 4F-treated Tsk^−/+^ mice.

Increased apoptosis is a one of many hallmarks of scleroderma [24]–[26]. Tsk^−/+^ hearts had more TUNEL positive cells than in C57Bl/6J hearts (Figure 4). 4F treatments significantly decreased the number of TUNEL positive cells in Tsk^−/+^ hearts compared to the number in untreated Tsk^−/+^ hearts but not to the level of TUNEL positive cells in C57Bl/6J hearts that actually had the least number of TUNEL positive cells (Figure 5).

### Endothelial Cell Up-Take of 4F

**Figure 6:**
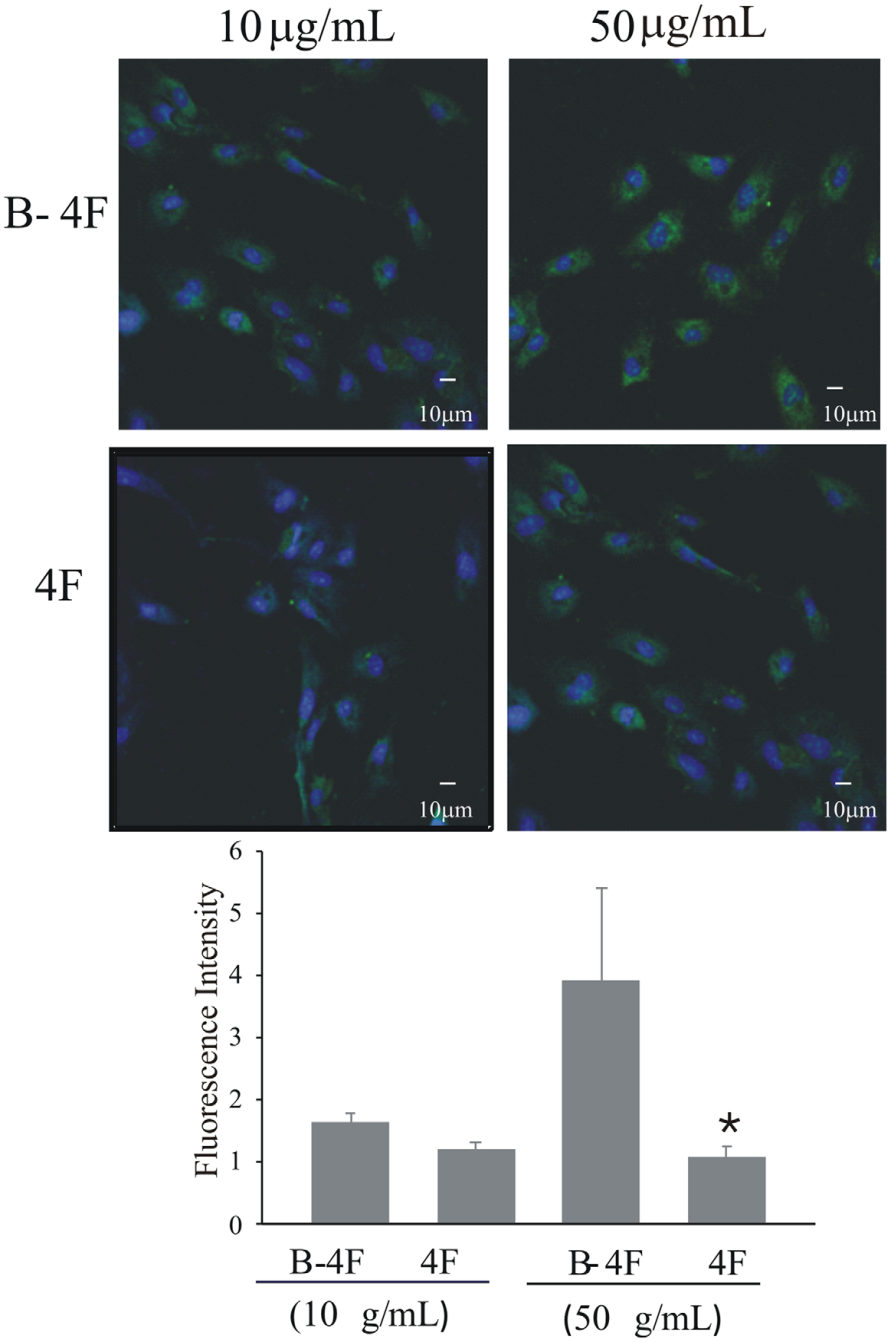
Cultured Endothelial Cells Take up 4F. EC cultures were treated with biotinylated 4F (B-4F) and unlabeled 4F. After 3 hours B-4F content is increased in ECs incubated with 10 and 50 µg/ml.

If 4F modulates IRF5 activation then it likely enters the cells to do so. Confocal microscopy shows greater fluorescence in EC cultures incubated with 10 and 50 µg/ml of B-4F than in EC cultures incubated with equal concentrations of unlabeled 4F (Figure 6). Image analysis of fluorescent intensity on a particle basis reveals that EC cultures incubated in media containing 50 µg/ml of B-4F was significantly increased 3.6 fold (p≤0.05) compared with the intensities in EC cultures incubated with unlabeled 4F. EC cultures incubated in media containing B-4F at 10 µg/ml concentration had an increase in fluorescence intensity that was approximately 30% more than the fluorescence in EC cultures in media containing unlabeled 4F.

### 4F-IRF5 Interactions

**Figure 7:**
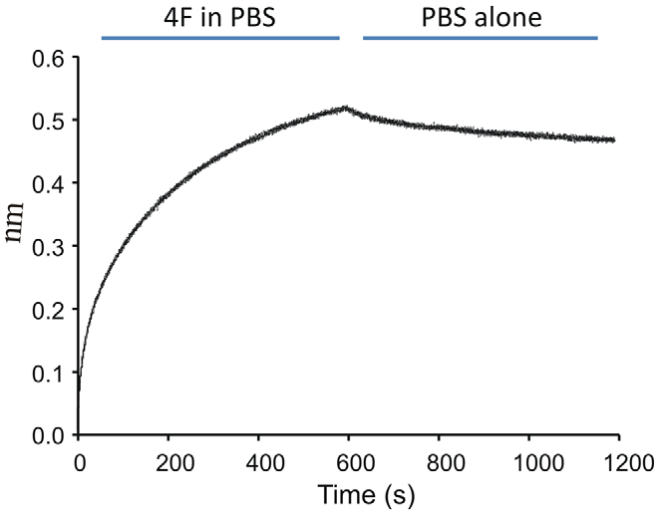
4F interacts with IRF5. Interactions of 4F with IRF5 were determined by biolayer interferometry. These curves show that the rate of 4F association with IRF5 is faster than their rate of dissociation.

**Figure 8:**
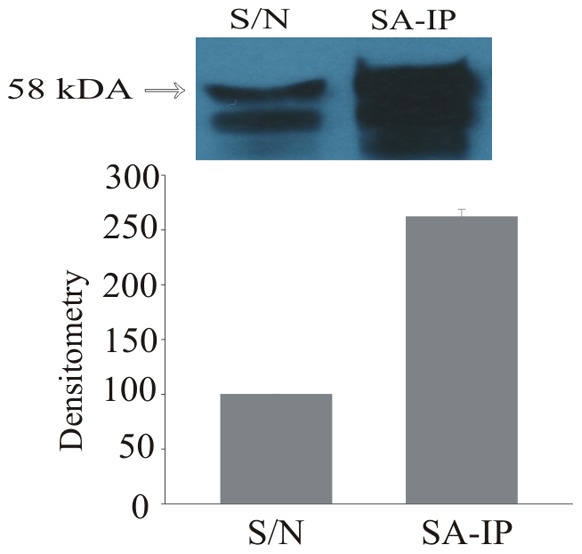
4F Interacts with IRF5 in EC Lysates. This Western blot analysis of B-4F pull-down assays were performed on EC lysates to which recombinant IRF5 (10 µg/mL, final concentration) was added. SA-agarose beads were used to pull down B-4F and associated proteins. In lane 2 is IRF5 bound to the B-4F precipitate is 2.5 fold higher than in control lane 1.

If 4F enters cells to modulate IRF5 expression and activation then it may also interact with IRF5. To determine if 4F interacts with IRF5 and to ascertain the nature of this interaction, we assessed rates of association and dissociation by biolayer interferometry. Our studies show that 4F rapidly associated with B-IRF5 bound to SA-biosensors. In contrast, dissociation of 4F from B-IRF5 on SA biosensors was slow. These data suggest that the interaction of 4F with IRF5 favors association over dissociation (Figure 7). Simple pulldown assays confirm that B-4F associates with IRF5 even in the presence of EC cytosolic proteins (Figure 8).

## Discussion

The purpose of this study was to investigate an alternative mechanism for 4F decreasing myocardial inflammation in Tsk^−/+^mice. While our previous report [3] focused on 4F’s ability to decrease inflammation by improving HDL function, the current study determines the effects of 4F on IRF5. We chose to investigate this potential mechanism for two important reasons. First, others have already shown that 4F decreases expression of TNFα, Il-6, and Il-1 [27], [28]. These inflammatory mediators are all gene products of IRF5 [9], [29]. Second, genetic links between IRF5 and SSc have been identified [30]. In this study we demonstrate that posterior wall thickness is significantly improved in Tsk^−/+^ mice after 4F treatment highlighting the reduction of fibrosis in the Tsk^−/+^ mice with 4F treatment even more. Together these reports provided the rationale for designing experiments to determine if 4F modulates IRF5 in the hearts of Tsk^−/+^ mice. Data from the experiments show that while IRF5 levels in Tsk^−/+^ hearts are comparable to their levels in control hearts, IRF5 in Tsk ^−/+^ hearts is activated, present in myocardial cell nuclei and that hearts from untreated Tsk^−/+^ have high levels of apoptotic cells. 4F treatments, on the other hand, decrease IRF5 expression, activation, nuclear translocation which begins to explain why hearts from 4F treated Tsk^−/+^ mice are less inflamed and have fewer apoptotic cells than hearts from untreated Tsk^−/+^ mice.

One of the reasons that we felt 4F decreased inflammation by a mechanism(s) other than those mediated by HDL function, was the extreme differences in the histopathology of the hearts from untreated and 4F treated Tsk^−/+^ mice. Untreated Tsk^−/+^ hearts contained much higher levels of oxidized phospholipids, and autoantibodies against oxidized lipids than control hearts [3]. In addition, untreated Tsk^−/+^ hearts were also more fibrotic and expressed more angiostatin than control hearts, which likely contributed to the notable losses in angiogenic potential in the hearts of these mice [3]. When Tsk^−/+^ mice were treated with 4F (1 mg/kg/d, 6–8 weeks), the treatment essentially eliminated oxidized phospholipids, and autoantibodies against oxidized phospholipids from the hearts. When the 4F-induced changes in myocardial inflammation in Tsk^−/+^ mice were examined in light of other studies showing that 4F works better when HDL is present [7], we began to consider the notion that this small amphipathic peptide inhibited mechanisms beyond those induced by proinflammatory HDL.

Although peptide mimetics and biologics have been used to target various mechanisms of disease, there is only one mimetic that we know of that specifically targets a transcription factor. COG_133_ is reported to possess anti-inflammatory properties, not because it binds oxidized lipids but rather because it binds and inactivates NFkappaB. The efficacy of COG_133_ was enhanced by linking the fragment to antennapedia to generate COG_112_, thereby increasing its cellular uptake [31]–[33]. In addition, COG_112_ inhibited NO production and iNOS expression in colonic epithelial cells [32]. Such observations lend support for the idea that small peptides may be useful tools for targeting transcription factors to inhibit oxidative stress.

In the current study, we showed that 4F enters EC in a concentration-dependent fashion even without a protein transduction domain. Our protein-protein interaction studies showed that 4F favors association over dissociation with IRF5 and that this interaction exists even in the presence of EC cytosolic proteins, where 4F must compete for access to IRF5. This observation however deserves an additional cautionary comment. We recognize that it is possible for 4F to interact with other cellular proteins, either in the membrane or in the signaling pathways mediating IRF5 expression and activation to indirectly down regulate IRF5. This possibility, and that 4F also has the potential to directly interact with IRF5 to decrease expression and activation will be investigated in much greater detail near future. Such studies are outside the scope of the current study.

Although there is overwhelming evidence in animal models that 4F improves HDL function and decreases atherosclerosis, our data are the first to suggest that 4F decreases inflammation by inhibiting IRF5 expression and activation. Therefore, our observations have important implications for future studies. First, they provide the rationale for designing agents that target IRF5 to protect against non-viral mediated inflammation of the heart. Second, IRF5 has the potential to preserve HDL function. Previous data showed a decrease of the atherogenic burden on HDL which might be associated with the inhibition of IRF5 [34]. Finally, as 4F appears to decrease myocardial inflammation by two distinct mechanisms they provide the rationale for developing agents that are specifically designed to distinguish between 4F’s effects on classical mechanisms of atherogenesis and those mediated by IRF5.

Although much is known concerning the cell biology of IRF5 in immune cells, little, if any, information is available concerning the role of IRF5 in the myocardium. This is likely a result of the fact that monocytes, myeloid cells and B-lymphoblasts express IRF5 many fold higher than the levels of IRF5 in the heart [35]. Thus, a potential limitation in the current study is the possibility that the changes we observed in myocardial IRF5 expression may actually result from changes in immune cell content. However, it should be noted that preliminary histology studies revealed that cells with IRF5 positive nuclei were myocardial in nature rather than immune and that cultured neonatal myocytes surprisingly expressed high levels of IRF5 that are also modulated by matrix in the same way as EC cultures [1], [36].

Our results are the first to demonstrate that IRF5 is activated and present in the nuclei of cells in the myocardium of an established murine model of myocardial inflammation and fibrosis. Our findings are also the first to show that 4F decreases myocardial expression of IRF5, a transcription factor that others have shown is central to host-defense and inflammation in a variety of autoimmune diseases [30], [36]–[38]. While our observations provide new insight into the anti-inflammatory properties of 4F, additional studies are required to determine how and why 4F alters the cell biology of IRF5 in Tsk^−/+^ mouse myocardium.

In conclusion, the current findings show that 4F decreases IRF5 levels and activation in the Tsk^−/+^ mouse hearts. Our observations demonstrate that 4F decreases myocardial inflammation by an alternative pathway in an established murine model of autoimmune disease. Our previous report and data here demonstrate that the mechanisms by which 4F decreases myocardial inflammation are multifactorial. Not only does 4F improve HDL function but it also targets IRF5 to decrease inflammation. Our results strongly suggest that developing specific inhibitors of IRF5 and/or their pathways of activation may result in new therapeutic modalities that would be capable of inhibiting inflammation and oxidative stress in autoimmune disease.
